# Withdrawal: Enhanced in vitro antiproliferative effects of EpCAM antibody-functionalized paclitaxel-loaded PLGA nanoparticles in retinoblastoma cells

**Published:** 2013-06-06

**Authors:** The Editors of Molecular Vision

Due to duplicate publication of part of Figure 1c from [1] in Figure 5 of [2], the authors of  have voluntarily withdrawn their article from Molecular Vision. The corresponding author of [2] was the first to bring this error to the attention of the Editors. He did so promptly, and it was he who first sought to withdraw the article.

## References

[1] Acharya S, Dilnawaz F, Sahoo SK (2009). Targeted epidermal growth factor receptor nanoparticle bioconjugates for breast cancer therapy.. Biomaterials.

[2] Mitra M Misra R Harilal A Sahoo SK Krishnakumar S Enhanced in vitro antiproliferative effects of EpCAM antibody-functionalized paclitaxel-loaded PLGA nanoparticles in retinoblastoma cells. Mol Vis 2011 17 2724 37Epub 2011 Oct 19 22065926 PMC3209422

